# MiRNA Regulatory Functions in Photoreceptors

**DOI:** 10.3389/fcell.2020.620249

**Published:** 2021-01-21

**Authors:** Julia Sophie Pawlick, Marta Zuzic, Giovanni Pasquini, Anka Swiersy, Volker Busskamp

**Affiliations:** ^1^Universitäts-Augenklinik Bonn, Department of Ophthalmology, University of Bonn, Bonn, Germany; ^2^Center for Regenerative Therapies Dresden (CRTD), Technische Universität Dresden, Dresden, Germany

**Keywords:** miR-182, miR-183, miR-124, retina, retinal degeneration, photoreceptors, rods, cones

## Abstract

MicroRNAs (miRNAs) are important regulators of gene expression. These small, non-coding RNAs post-transcriptionally silence messenger RNAs (mRNAs) in a sequence-specific manner. In this way, miRNAs control important regulatory functions, also in the retina. If dysregulated, these molecules are involved in several retinal pathologies. For example, several miRNAs have been linked to essential photoreceptor functions, including light sensitivity, synaptic transmission, and modulation of inflammatory responses. Mechanistic miRNA knockout and knockdown studies further linked their functions to degenerative retinal diseases. Of note, the type and timing of genetic manipulation before, during, or after retinal development, is important when studying specific miRNA knockout effects. Within this review, we focus on miR-124 and the miR-183/96/182 cluster, which have assigned functions in photoreceptors in health and disease. As a single miRNA can regulate hundreds of mRNAs, we will also discuss the experimental validation and manipulation approaches to study complex miRNA/mRNA regulatory networks. Revealing these networks is essential to understand retinal pathologies and to harness miRNAs as precise therapeutic and diagnostic tools to stabilize the photoreceptors’ transcriptomes and, thereby, function.

## Introduction

**FIGURE 1:**
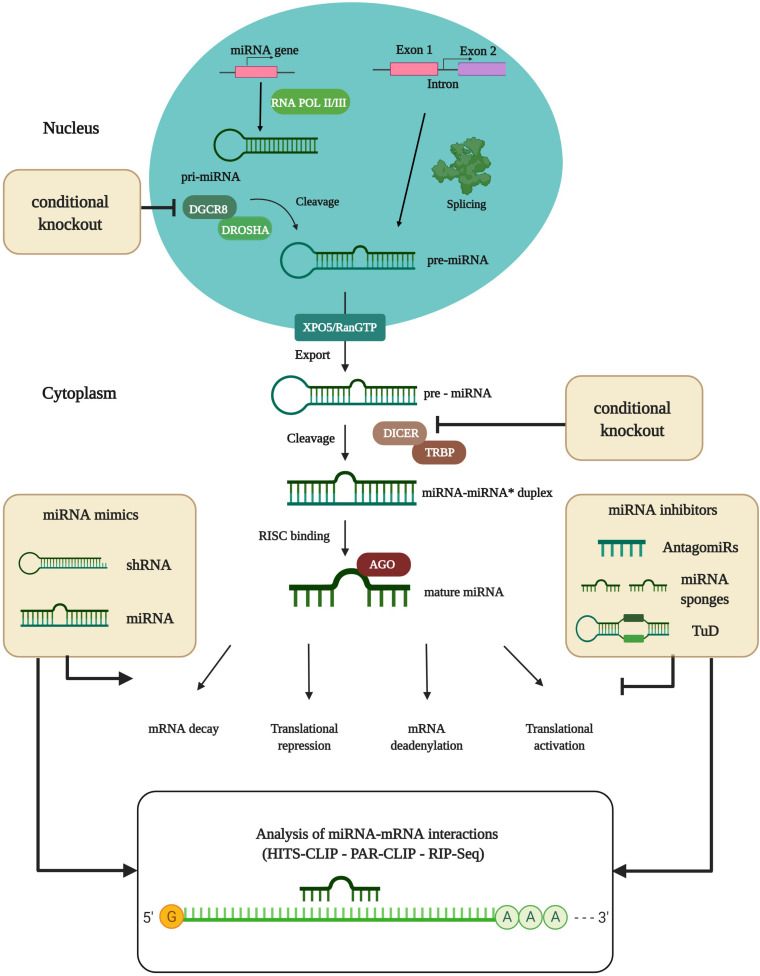
MiRNA biogenesis and experimental analysis of miRNA-mRNA interactions. DNA sequences encoding for miRNAs are transcribed into primary-miRNAs (pri-miRNAs) by partially complementary RNA polymerase II/III. Pri-miRNAs are subsequently cleaved by the miRNA-processing complex consisting of Drosha ribonuclease and the double-stranded RNA binding domain partner protein DGCR8 that form precursor-miRNAs (pre-miRNAs). The Drosha/DGCR8 complex and Dicer endoribonuclease are used for conditional knockout studies (cKO) to identify the actions of particular miRNAs. Full knockout of miRNA-processing machinery has proved lethal. The pre-miRNA is then transported into the cytoplasm by the exportin (XPO5)/RanGTP complex. In the cytoplasm, the Dicer/*trans-*activation response RNA binding protein (TRBP) nuclease complex cleaves the pre-miRNA, thereby producing the miRNA duplex. Finally, the duplex is loaded onto the Argonaute (AGO) protein as a part of the RNA-induced silencing complex (RISC) where one of the strands is removed. The remaining strand remains bound to the AGO protein, which is now ready to target mRNA. Translational efficiency is thereby reduced, mainly as a consequence of mRNA cleavage or deadenylation. Alternatively, miRNA biogenesis can proceed *via* splicing events where Drosha cleavage is replaced. MiRNA pathways can be modulated by miRNA mimics or inhibitors. Analysis of miRNA-mRNA interactions is done by RNAse digestion of AGO proteins, combined with next-generation sequencing (NGS) techniques like HITS-CLIP or PAR-CLIP, or novel techniques like RNA immunoprecipitation combined with NGS (RIP-Seq). shRNA, small hairpin RNA; TuD, Tough decoy. Figure was created with BioRender.com.

MicroRNAs (miRNAs) are small, non-coding RNAs, acting as quantitative regulators of gene expression, which are characterized by an average length of 22 nucleotides (nt) (Ghildiyal and Zamore, 2009; Ha and Kim, 2014). MiRNAs were discovered in 1993 in the nematode *Caenorhabditis elegans* (Lee et al., 1993). The biogenesis of miRNAs is divided into several steps (Figure 1). DNA sequences encoding for miRNAs are transcribed into primary miRNAs (pri-miRNAs) by RNA polymerases II/III (Lee et al., 2004; Borchert et al., 2006). Pri-miRNAs build hairpin-like structures or stem-loops by self-annealing. These structures are cleaved 11 base pairs from the hairpin stem by the miRNA-processing complex, consisting of Drosha ribonuclease and the double-stranded RNA binding domain partner protein DiGeorge critical region 8 (DGCR8): this forms the precursor miRNA (pre-miRNA), consisting of a 70-nt-long sequence and a 5′ phosphate and 2-nt overhang at the 3′ end (Lee et al., 2002; Ha and Kim, 2014). For the last step of miRNA maturation, the pre-miRNA is exported into the cytoplasm by the exportin-5 (XPO5)/RanGTP complex (Yi et al., 2003; Lund et al., 2004). At this point, Dicer endoribonuclease and its *trans-*activation response RNA-binding protein (TRBP) cleave the pre-miRNA and add a 5′

phosphate and a new 2-nt 3′ overhang by cutting the pre-miRNA ∼22-nt from the cleaving site of the miRNA processing complex. This step gives rise to the miRNA duplex (Lee et al., 2003). Finally, the Argonaute (AGO) protein, as a part of the RNA-induced silencing complex (RISC), binds to the miRNA and removes one of the strands. The remaining strand is bound to the AGO protein and ready to bind partially-complementary mRNA transcripts. MiRNA biogenesis can also occur through alternative pathways, where Drosha cleavage is replaced and the miRNAs are processed *via* splicing events when miRNAs reside in the introns of protein-coding genes (Westholm and Lai, 2011). The AGO proteins, as core components of the RISC complex, have a coordinating function for localizing mRNA transcripts, and are therefore essential for correct miRNA function (Bartel, 2009; Ha and Kim, 2014). A single miRNA can have thousands of *in silico* annotated target mRNAs, regulating multiple genes which often participate in the same biochemical pathway. This is due to similarities in the 3′ untranslated region (3′ UTR) of specific mRNAs which are bound by miRNAs (Lewis et al., 2005). However, experimental *in vivo* validations only result in very few mRNA targets upon miRNA manipulation (Rojo Arias and Busskamp, 2019). The RISC components are the most obvious targets for regulation. Nevertheless, other proteins that take over positive or negative control of miRNA effects have also been identified. Most miRNAs are translational repressors, or promote deadenylation and decay of mRNAs. However, miRNAs can also act as activators of translation by switching AGO2 from a repressor to an activator (Vasudevan and Steitz, 2007; Vasudevan et al., 2007). In general, miRNAs have strong regulatory properties in many biological systems, including the eye, whilst regulating the transcription of genes in cells to maintain their homeostasis and function (Sundermeier and Palczewski, 2016). Hence, their dysregulation, especially during development, can lead to diverse pathological conditions like genetically-inherited disorders, neurodegenerative diseases, and cancer, as well as autoimmune and cardiovascular diseases (Ha, 2011; Dong et al., 2015; Chu-Tan et al., 2018). It is thought that miRNA regulatory networks provide robustness to biological systems whose faultless functioning is constantly being endangered by external and internal interferences (Ebert and Sharp, 2012). Approximately 25% of the human miRNA genes are structured in clusters. Their expression is normally tightly regulated, but altered in pathologies. Of note, epigenetic modifications have been reported that change the expression levels of proteins which are important for miRNA biogenesis (Kabekkodu et al., 2018). In addition, it has been reported that accelerated turnover of miRNAs depends on the activity of photoreceptors, i.e., their exposure to light (Krol et al., 2010a). Clusters are expressed as a polycistronic transcript, with a high sequence homology between the members. In general, clusters of miRNAs are formed by several miRNA genes that are located next to each other on the chromosome. The genes are transcribed as one long pri-miRNA, which is then processed into several individual pre-miRNAs (Altuvia et al., 2005). Each of the miRNAs has small differences in their seed sequence, leading to different mRNA targets (Dambal et al., 2015). However, if the seed sequences have diverged and their mRNA targets have diversified, they cooperate by targeting different genes in common pathways, which amplifies the downstream effects. The cooperative work of multiple miRNAs to target multiple functionally-related genes enables coordinated control of gene networks (Na and Kim, 2013). However, there are similarities in their binding properties to target mRNAs and they can compensate for each other’s function (Jin et al., 2009).

Photoreceptors are the cells in the body with the highest metabolic activity, and are subject to high levels of external stress (Sung and Chuang, 2010). In this context, miRNAs play an important role in the functioning and survival of photoreceptors (Sundermeier and Palczewski, 2016). The fact that not all retinal diseases are linked to specific genes supports the idea that the dysregulation of certain miRNAs can cause the progression of retinal disorders. Experimental disruption of the miRNA processing machinery can lead to the loss of cone outer segments in humans, triggering their dysfunction, resulting in blindness (Busskamp et al., 2014). Furthermore, introducing gene-trap constructs downstream of a gene, expressing the miRNA cluster miR-182/96/183 that is highly expressed in the retina, leads to its inactivation and to progressive synaptic defects (Lumayag et al., 2013). Moreover, it has been shown that miR-124, as the most abundant miRNA in the brain, plays an important role in the progression of neovascular and atrophic form of age-related macular disease (AMD) (Chu-Tan et al., 2018). In this context, mimics of miR-124 in the eye decreased the inflammatory response in both forms of AMD, improving overall retinal function. Therefore, revealing miRNA regulatory pathways in retinal cell types is crucial to obtain a better understanding of the molecular processes that lead to ocular diseases for which there is currently no treatment. In addition, the investigation of retinal miRNAs provides a basis for developing therapies to significantly improve the quality of life of affected patients. Here, we will emphasize the role of the highly-abundant miR-182/96/183 cluster and miR-124 within the retina. We will discuss how sophisticated experimental studies have revealed miRNA functions within different retinal cell types.

## miRNA Regulation in the Retina

Photoreceptor cells and retinal pigmented epithelium (RPE) cells are characterized by high rates of metabolism and protein synthesis. In addition, they are constantly exposed to toxic by-products of phototransduction. Moreover, they have to stay viable and functional under highly oxidizing conditions. All these processes cause high levels of stress to the cells, making them much more vulnerable to precocious death (Sundermeier and Palczewski, 2016). Acting as fine tuners of gene expression, miRNAs take over important functions with respect to photoreceptor survival and function (Sundermeier and Palczewski, 2016). On the other hand, *in vivo* and *in vitro* studies have shown that there are several miRNAs that are potentially associated with the cellular processes that lead to AMD (Wang et al., 2014; Chu-Tan et al., 2018). Investigating the miRNA transcriptome (miRNome) of retinal cells is the first step toward revealing miRNA regulatory pathways in health and disease. Studies have been conducted on the human and mouse miRNomes to reveal differences and similarities in miRNA regulatory pathways between human and mouse eye (Karali et al., 2010, 2016). In particular, research has focused on the most conserved miRNAs. This comparison is indispensable, because these two organisms show essential differences in the structure and function of the eye: however, mouse models are used more in vision research. These studies have revealed that one third of the retinal miRNAs expressed in human samples are also expressed in the mouse retina (Karali et al., 2016). Hence, mouse models are well-suited model systems for retinal miRNome studies for miRNAs that have been found to be expressed in both species. Almost a fifth of all known miRNAs are expressed in the retina, and a limited set of these miRNAs has been identified as playing an important role in the development and function of the retina (Krol et al., 2010b; Lumayag et al., 2013). This set consists of miR-182-5p, miR-183-5p, and miR-124-3p, as well as miR-96-5p and miR-9-5p. Moreover, miRNA expression is tissue specific and its regulation changes, depending on the developmental stage. This demonstrates that miRNAs are involved in important retinal maturation processes, and that their expression pattern is tightly controlled (Lagos-Quintana et al., 2002). The misregulation of miRNA expression is therefore a proximate cause of retinal degeneration and disease (Damiani et al., 2008; Arora et al., 2010; Georgi and Reh, 2010; Busskamp et al., 2014; Sundermeier et al., 2014; Ohana et al., 2015).

The length and sequence of mature miRNAs is highly heterogeneous: this is different from the canonical miRNA sequence (Morin et al., 2008). As a result, one miRNA can have several variants, called isomiRs, that are characterized by addition or deletion of nucleotides at the 3′ and/or 5′ end of the miRNA and/or substitutions within the sequence (Landgraf et al., 2007; Morin et al., 2008; Martí et al., 2010). Most miRNA-mRNA interactions are based on the binding of the miRNA seed sequence to its target mRNA (Helwak et al., 2013). Nucleotide substitutions at the 5′ end of the miRNA result in a modified seed sequence, resulting in a changed target specificity and far-reaching effects on miRNA functionality (Cammaerts et al., 2015). In the isomiR variant of miR-124-3p, a single nucleotide substitution in the seed region resulted in a change in its target specificity, when comparing with the canonical miRNA specificity (Karali et al., 2016). This resulted in an altered gene regulatory property and showed that gene regulation within the retina is complex but also necessary to ensure proper tissue function. The miR-124-3p and miR-183-5p isomiRs accounted for a large part of the retinal miRNome analysis (Karali et al., 2016). During miRNA biogenesis, either the 5′ or 3′ arm of the miRNA duplex is favorably cleaved by Drosha and Dicer: this becomes the mature miRNA (Khvorova et al., 2003; Schwarz et al., 2003). Still, next-generation sequencing (NGS) data have revealed that both arms are cleaved and detectable (Yang et al., 2011; Li et al., 2012; Neilsen et al., 2012; Zhou et al., 2012; Kang et al., 2013). The mature miR-183, miR-182, and miR-96 have almost identical seed sequences (Dambal et al., 2015). A single base difference in the seed sequence of miR-182 and miR-96 changes the binding property to the mRNA target sequence (Jalvy-Delvaille et al., 2012; Li et al., 2014). Nevertheless, their targets often lie in the same pathways, facilitating that these miRNAs control several parts of a cellular process (Dambal et al., 2015).

Disrupting miRNA regulatory pathways during development can have severe effects, such as aberrant photoreceptor layer architecture and progressive photoreceptor degeneration (Georgi and Reh, 2010). MiRNAs have been found to control transcription factors like *Pax6*, which is expressed in a spatiotemporal pattern in different tissues, including the developing retina, lens, cornea, and mature ocular cell types, during development (van Heyningen and Williamson, 2002; Kaspi et al., 2013). Analysis of the *Pax6* 3′ UTR has revealed that cooperative miRNA regulation of *Pax6* mediates developmental control and fine tuning of *Pax6* levels during development (Ryan et al., 2018). Changes in miRNA expression have also been investigated in a retinal degeneration model in which retinal damage was induced by light (Saxena et al., 2015). Transcriptomic analysis revealed that a large set of miRNAs regulates the immune response connected to the light-damage changes. This supported the theory that miRNAs play an important role in retinal degenerative diseases that are characterized by acute retinal damage (Veleri et al., 2015). In AMDs, miRNAs are often associated with the regulation of inflammatory processes which highlights the need for a better understanding of miRNA regulatory networks (Rutar et al., 2010; Chu-Tan et al., 2018).

Müller glia (MG) are the predominant glia in the retina: they nurture and protect retinal neurons, maintain the homeostasis of the retina, and support structural integrity (Bringmann et al., 2006). Consequently, the loss of mature MG can lead to impairment of the retinal structure (Byrne et al., 2013). Neuronal loss leads to retinal remodeling, a process in which MG expand and fill the neuronal gaps. They form a glial scar, which is a major limiting factor regarding transplantation approaches to restore retinal function (Jones and Marc, 2005; Reh, 2016). To study the role of miRNAs in MG function, Dicer1 was specifically deleted in MG (Wohl et al., 2017). Here, it is of great importance that knockouts of the miRNA processing machinery are conditional (cKO) because full deletion during embryonic development in mouse models is lethal (Bernstein et al., 2003; Fukuda et al., 2007; Morita et al., 2007; Wang et al., 2007). The deletion of Dicer1 led to a significant decline in those miRNAs, called mGliomiRs, that are highly expressed in MG (Wohl and Reh, 2016b). The decline in MG miRNAs led in early phases to an increased number of MG, and in MG migration toward the outer nuclear layer. At later stages, glia accumulations and the deformation of the retinal architecture were found. A key player in this process was the miRNA miR-9 that targets the extracellular matrix molecule Brevican (encoded by *Bcan*). All these results led to the conclusion that miRNAs play an important role in MG function, which is required for the maintenance of retinal structure and function (Wohl et al., 2017). Moreover, overexpression of neuronal (Wohl and Reh, 2016a) or retinal progenitor miRNAs, in combination with inhibition of MG miRNAs (Wohl et al., 2019), can reprogram MG into late retinal progenitor cells that differentiate into bipolar-like neuronal cells. This suggests that miRNAs are involved in MG-reprogramming tool for retinal regeneration. Another Dicer conditional knockout mouse model was used to identify which miRNAs are important for retinal development (La Torre et al., 2013). Three different miRNAs, let-7, miR-125, and miR-9 were found to act as regulators, by changing the competence of retinal progenitor cells. In addition, the overexpression of these miRNAs accelerated retinal development. Other studies have investigated miRNA functions in cone photoreceptors, which are indispensable for daylight and high-acuity vision. Cone photoreceptor-specific miRNA-deficient mice showed a gradual depletion of DGCR8 protein over time, leading to a progressive loss of cone outer segments and low sensitivity to high light levels (Busskamp et al., 2014). Besides neurodegenerative retinal diseases, developmental genetic disorders such as microphthalmia, anophthalmia, and coloboma (MAC) cause structural eye malformations: a heterozygous mutation in the seed region of miR-204 has been described in MAC patients (Conte et al., 2015). Seed sequence modifications impact on the mRNA targets, resulting in photoreceptor alterations, reduced numbers of rod and cone photoreceptors, and increased levels of apoptosis. These findings highlight the important function of miR-204 during retinal development.

## The miRNA Cluster 182/96/183 in Photoreceptors

**FIGURE 2:**
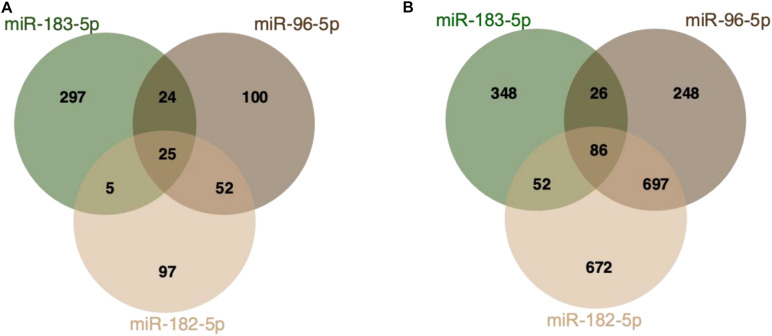
MiR-182/96/183 cluster target interactions. **(A)** Venn Diagram displaying miRTarBase entries for miR-182/96/183 cluster targets (Chou et al., 2018). All three miRNAs share 25 validated common targets. **(B)** Predicted miR-182/96/183 targets based on TargetScan (release 7.2) (Agarwal et al., 2015). The analysis is based on analyzing the presence of target sites that match the seed region of each miRNA. All three miRNAs share 86 predicted biological targets.

MiR-182/96/183 is a sensory-neuron enriched miRNA cluster. It is highly and prevalently expressed in mature photoreceptors and in the inner nuclear layer (INL) of the retina (Xu et al., 2007). In particular, miR-182 and miR-183, and also miR-96, play an important role in the maintenance and function of cone outer segments (Busskamp et al., 2014). MiR-96 also plays a major role in the cells of inner ear hairs (Lewis et al., 2009; Mencía et al., 2009). MiR-182, miR-96, and miR-183 are co-expressed on a single primary transcript and share high sequence homology, suggesting overlapping, but unique functions (Xu et al., 2007; Figure 2). As mentioned before, clusters can compensate for each other’s function, which has been shown by targeted deletion of the miR-182 (Jin et al., 2009). Here, no changes in phenotype were observed, indicating that miR-183 or miR-96 had very likely a compensatory effect. In general, the cluster is responsible for global regulation of many downstream genes that are involved in several pathways such as synaptogenesis, synaptic transmission, and photoreceptor functions (Lumayag et al., 2013; Busskamp et al., 2014) and it has a protective effect on neurons by targeting Caspase-2 (Casp-2) (Zhu et al., 2011).

The expression of mature cluster miRNAs is low early in development, but increases after birth and is most abundant in the adult retina (Xu et al., 2007). The pri-miR-183/96/182 is highly expressed early in development but, due to reduced enzymatic processing, the expression of the mature miRNA cluster is delayed and dependent on the developmental stage. An interplay between a long, non-coding RNA [lncRNA Rncr4 (retinal non-coding RNA 4)] and the miRNA cluster has been described: this is crucial for postnatal retinal development (Krol et al., 2015). An enforced expression of mature miR-182/96/183 early in development can have negative effects on the morphology of the retinal layers. The regulation of miRNA cluster expression is indispensable for the correct development and function of the retina. Moreover, the miRNA cluster plays an important role in the formation of tight conjunctions between MG cells and photoreceptors (Krol et al., 2015). The cluster was found to have a dynamic diurnal expression pattern suggesting that its regulation is coupled to the circadian rhythm (Xu et al., 2007). Later, it was shown, that the miRNA cluster is reversibly up- and downregulated in the retina *in vivo* during light-dark adaption, independent of the circadian rhythm (Krol et al., 2010a). Additionally, re-expression of miR-182 and miR-183 prevented cone photoreceptor function loss *in vivo*, even after the miRNA processing machinery was disrupted. *In vitro*, the administration of the two miRNAs led to the formation of inner segments, connecting cilia, and short outer segments in stem-cell-derived 3D retinal organoids. In this way, the photoreceptors became light sensitive (Busskamp et al., 2014). Another study demonstrated that the cluster is an important regulator of *PAX6* and that it is important for retinal tissue morphogenesis (Peskova et al., 2020). To inhibit the cluster in organoid forming human pluripotent stem cells (hPSCs), a tough decoy approach was used. Also, abnormalities were observed in retinal organoid morphology, together with an upregulation of neuron- and retina-specific genes. A single knockout of miR-182 in mouse models did not lead to any significant changes in retinal architecture. However, deletion of both miR-183 and miR-96 caused defects in cone maturation (Xiang et al., 2017) linked to their target Slc6a6, a taurine transporter, which is needed for the maturation and maintenance of photoreceptors. The formation of correct synaptic connections between photoreceptors and postsynaptic retinal cells was also shown to be miRNA-dependent. A knockout mouse model generated from an embryonic stem cell clone (ESC), where a gene trap was inserted downstream of the first exon of the miR-182/96/183 gene, resulted in progressive synaptic defects in photoreceptors, and progressive retinal degeneration (Lumayag et al., 2013). MiR-182 has also been reported to impact axonal growth of retinal ganglion cells in *Xenopus laevis* (Bellon et al., 2017).

So far, only a handful of the thousands of annotated cluster miRNA targets have been experimentally validated in retinal cell types. As important biological processes fall under their regulation, further research in the coming years will provide deeper insights revealing their functions in health and disease. In summary, the miR-182/96/183 cluster is indispensable for proper retinal development and function, such as maintaining photoreceptor outer segments, synaptogenesis, and axonal growth. Therefore, it might be possible to use these miRNAs as therapies to cure neurodegenerative retinal diseases.

## miR-124 and Its Role in Neurodegeneration and Neuronal Differentiation

miR-124 is one of the most abundant miRNAs in the brain, accounting for 25% of all brain miRNAs (Lagos-Quintana et al., 2002). It is also highly expressed in the retina, where it supports the maturation of photoreceptors. In addition, the partial loss of miR-124 during development leads to reduced opsin expression and cone photoreceptor death (Sanuki et al., 2011; Karali et al., 2016). This miRNA has three paralogs with six genomic copies. A complete miR-124 knockout has been generated in human induced pluripotent stem cells (hiPSC) that were subsequently differentiated to neurons and analyzed: loss of miR-124 led to morphological and functional alterations, as well as different neurotransmitter profiles and decreased long-term viability. However, the initiation of neuronal differentiation was independent of miR-124 (Kutsche et al., 2018). This finding was a bit surprising, as the overexpression of miR-124 in cell lines and embryonic stem cells mediates neuronal differentiation (Krichevsky et al., 2006; Makeyev et al., 2007); overexpression of miR-9 and miR-124 in human fibroblasts causes them to differentiate into neurons (Yoo et al., 2011). Additionally, in HeLa cells, delivery of miR-124 duplexes caused acquisition of neuronal gene profile (Lim et al., 2005). Because of its important role in central nervous system including retinal neurons, miR-124 dysregulation is connected to certain diseases, including Alzheimer’s (AD), Parkinson’s, and AMD (Smith et al., 2011; Wang et al., 2014; Sun et al., 2015). In the degenerating retina, miR-124 expression and its cellular location are altered in human and rodent tissues (Chu-Tan et al., 2018). It has been shown that miR-124 targets mRNAs which code for chemokines that are upregulated in neovascular and atrophic forms of AMD when physiological miR-124 levels are decreased. Intravitreal delivery of miR-124 reduced the chemokine expression levels, highlighting its anti-inflammatory properties. Photoreceptor death could be reduced, and overall retinal function was improved (Newman et al., 2012; Chu-Tan et al., 2018). The activation of the innate immune system is connected to the pathogenesis of certain retinal degenerative diseases, for example AMD or diabetic retinopathy (DR) (Edwards et al., 2005; Hageman et al., 2005; Hou et al., 2015). In the case of DR, elevated levels of monocyte chemotactic protein-1 (MCP-1) can be detected in tear fluid (Liu et al., 2010) and vitreous fluid (Wakabayashi et al., 2011; Chernykh et al., 2015). The same work showed that miR-124 takes over an anti-inflammatory role by targeting the 3′ UTR of the MCP-1 gene, therefore decreasing MCP-1 expression and inflammation (Dong et al., 2015). Altogether, anti-inflammatory properties of miR-124 have an impact as a therapeutic for treating retinal degenerative diseases (Chu-Tan et al., 2018).

## Other Key Photoreceptor miRNAs

Other miRNAs, like the miR-181a and miR-181b have also been shown to control the expression of genes that are involved in mitochondrial biogenesis and function in the retina (Indrieri et al., 2019). In this regard, downregulation of these miRNAs increased mitochondrial turnover, thereby protecting photoreceptors from degeneration. Additionally, miR-181a and miR-181b are highly expressed in the retina, notably in retinal ganglion cells (RGCs), inner cell layers and in brain areas that are related to visual function (Ryan et al., 2006; Kapsimali et al., 2007; Karali et al., 2007). Furthermore, these two miRNA species represent about 17% of the cone photoreceptor miRNome (Busskamp et al., 2014). Gain- and loss-of-function approaches on these two miRNAs revealed that they impact on axonal growth and specification of retinal cells by fine tuning of the MAPK/ERK pathways (Carrella et al., 2015). Their expression is crucial for the formation of neural connections in the retina (Carrella et al., 2015). Also, miR-204 has been found to be expressed in photoreceptors and plays an important role in retinal development (Conte et al., 2015). In this connection, a single heterozygous point mutation was analyzed in miR-204, which is the only known miRNA mutation that causes inherited retinal dystrophy in humans (Conte et al., 2015). This mutation is within the miR-204 seed region and leads to an autosomal dominant phenotype. This mutation may impact as a loss of function, resulting in non-recognizable wild type target genes or as a gain of function *via* new unconventional targets of miR-204. The therapeutic potential of miR-204 has been investigated by subretinal delivery of AAVs carrying the miR-204 pre-miRNA (Karali et al., 2020). The administration led to a decrease in apoptosis of photoreceptors and microglia activation in mouse models displaying inherited retinal diseases. Due to this neuroprotective function, the use of miR-204 as a therapeutic agent represents a promising mutation-independent approach for curing forms of blindness.

## Sophisticated Approaches to Study miRNA Regulatory Pathways

Studying the miRNome of tissues that consist of different cell types, like the retina, can be challenging. For technical reasons regarding the detection of these small RNA molecules, only highly abundant miRNAs have been studied so far. *In vivo*, a high heterogeneity of neuronal cell types and progenitors may falsify the results of studying specific miRNA expression in defined cell types, due to the differences in coding and non-coding transcriptomes (Yaworsky and Kappen, 1999). This can lead to an insufficient view, and misinformation about the miRNome of specific cell types. Pooling neuronal samples to obtain sufficient material for transcriptomic studies masks cell-type-specific miRNomes and their target mRNAs. Moreover, miRNA regulatory networks can be complex, as they have a large number of targets including non-canonical binding events (Chi et al., 2012; Moore et al., 2015). The ongoing technological development and refining of assays facilitate more precise studies, providing consistent and reliable results. Labeling cells with cell-type-specific markers allows the isolation and a more narrowed miRNome analysis on a homogenous cell population. This has been achieved by using transgenic approaches, where green fluorescent protein (GFP) was exclusively expressed in mouse cone photoreceptors (Fei, 2003). Moreover, it is important to consider the developmental stage, especially when using animal models, because of changes in the expression of specific miRNAs as mentioned before (Xu et al., 2007; Krol et al., 2015). To investigate the effects of missing post-transcriptional gene regulation by miRNAs, *in vivo* and *in vitro* knockdown studies of the miRNA machinery and of certain miRNAs has been performed (Sanuki et al., 2011; Busskamp et al., 2014). In this context, the time point of manipulation is important in order to interpret the obtained results. It was shown that DGCR8 deletion had a time-delayed effect (Busskamp et al., 2014) due to the high stability of the DGCR8 protein, likely because of its phosphorylation and interactions with other proteins (Han et al., 2009; Herbert et al., 2013; Cheng et al., 2014). Hence, the effects of missing miRNAs could only be seen after postnatal day 30, with a fully developed retina, leading to an incomplete view of the impact of the missing miRNA processing machinery during development (Busskamp et al., 2014). Recent developments in genomic engineering have also facilitated the generation of complete miRNA knockouts, such as deleting all six miR-124 alleles by clustered regularly interspaced short palindromic repeats (CRISPR/Cas9). Thereby, it became obvious that also other miRNAs took over the regulatory space in absence of miR-124. The knockout of a highly abundant miRNA species is not leading to a vacuum of miRNA regulation and a sophisticated interpretation of the phenotype requires also to study effects of *de novo* upregulated miRNAs in the cells of interest. Hence, in comparison to genes, studying miRNA knockout effects is more complex and requires sophisticated system level analysis (Kutsche et al., 2018). Chemically-engineered oligonucleotides termed “AntagomiRs” are used for silencing endogenous miRNAs (Krützfeldt et al., 2005). This approach has been used for down-regulation studies of endogenous miR-124 (Cao et al., 2007; Visvanathan et al., 2007; Cheng et al., 2009; Åkerblom et al., 2012). Here, the results turned out to be controversial, suggesting that antisense nucleotides trigger only transient inhibition, and that the knockdown is not sufficient. This was seen especially in progenitor cells, as their high proliferation rate affected their efficacy. Moreover, so-called miRNA sponges were used to analyze effects of miRNA silencing on cellular processes. These miRNA sponges, holding multiple tandem binding sites to a miRNA of interest, are expressed from strong promoters and bind specifically to miRNA seed families. Nevertheless, their silencing efficacy is comparable to approaches using antisense nucleotides (Ebert et al., 2007). Interestingly, sponge cassettes have been delivered to specific retinal cell types by adeno-associated viruses (AAVs) to analyze miRNA actions in neuronal cells (Krol et al., 2010a) as well as in transgenic mouse models (Zhu et al., 2011).

Another approach to study miRNA functions focused on the robust and simultaneous suppression of different pairs or groups of miRNAs that are not related to each other (Hollensen et al., 2013). So-called “Tough Decoy” (TuD) inhibitors were designed that are characterized by hairpin structures carrying two or more miRNA recognition sites. TuD allows to suppress several miRNAs *via* one DNA-encoded RNA inhibitor, making them a valid approach for suppression studies of miRNA clusters or families. Yet, TuD design by predicting target mRNAs is challenging because miRNAs bind to their messenger RNAs by base pairing with 6–8 nucleotides only (Chi et al., 2009). In this respect, the biochemical isolation of AGO proteins with RNAse digestion, combined with next-generation sequencing (NGS) techniques such as HITS-CLIP and PAR-CLIP, have been developed to analyze miRNA-mRNA pairs (Chi et al., 2009; Tan et al., 2013). Novel techniques that do not rely on crosslinking for isolating miRNAs and mRNAs, such as RNA immunoprecipitation combined with NGS (RIP-Seq), have also helped to increase our understanding of the miRNA targetome in neurons (Malmevik et al., 2015). Thereby, the RIP-seq technique brings the advantage that the AGO protein is in direct contact with miRNAs and mRNAs within the RISC complex, providing the opportunity to take a snapshot of the ongoing gene-regulatory processes in a cell to analyze biologically active miRNAs and their targets. However, when analyzing miRNA/mRNA targets with low- or high throughput molecular assays, it should be noted that although the binding of the miRNA to the mRNA actually takes place, it does not result in a change of the macroscopic phenotype, thus having no biological effect (Pinzón et al., 2017). In this context, genome editing tools have helped to probe and validate miRNA/mRNA interactions in the last years, that evoke a change in the phenotype (Bassett et al., 2014). Further experimental validations such as luciferase reporter assays are indispensable and vitally important to analyze *in silico* predicted miRNA/mRNA interactions (Ko et al., 2009; Jin et al., 2013). Luciferase reporter assays are used in order to analyze, if miRNAs bind to the 3′ UTR of their target genes (Ko et al., 2009). Ultimately, the impact of miRNA regulation must also be studied at the protein level using highly sensitive quantitative techniques. Commonly used miRNA target prediction programs rely on the molecular rules of RISC/target binding (Mockly and Seitz, 2019). Computational algorithms have shown to be the driving force of predicting miRNA targets (Bentwich, 2005; Rajewsky, 2006; Doran and Strauss, 2007; Mazière and Enright, 2007). It is based on the programming alignment to identify the 3′ UTR and the complementary miRNA seed sequence to predict miRNA-mRNA interaction. Still, evidence suggests that these predicted interactions do not necessarily have a functional role (Didiano and Hobert, 2006). For instance, there is a clear discrepancy between predicted and validated miR-182/96/182 targets (Figure 2). Although the predictions of the targetome and genetic networks regulated by individual miRNAs are becoming more and more reliable, the interaction of different miRNAs must be taken into account to draw meaningful conclusions about biological effects of miRNAs on mRNA and protein levels (Rojo Arias and Busskamp, 2019). Therefore, it is indispensable to also validate physiologically relevant targets of miRNAs experimentally (Kuhn et al., 2008).

## Conclusion

miRNAs impact on retinal development and function, especially on the survival and maintenance of photoreceptors. Therefore, it is not surprising that their misregulation is linked to various retinal degenerative diseases, as well as developmental genetic disorders. Increasing our knowledge of miRNAs is of great importance: to date, however, studies on miRNA regulatory networks are rare due to the complexity of the experimental procedures for small RNAs. Furthermore, these experiments require large amount of tissue samples, are expensive, and are limited in their application. The manipulations of miRNA regulatory networks are not trivial: the timing of the manipulation plays a crucial role as well. In addition, the knockout of highly abundant miRNAs results in other miRNAs taking over the regulatory space, which impedes a proper interpretation of the results for the manipulated miRNAs. This is especially important given the high annotated number of mRNA targets for any given miRNA species, because most studies to date have only experimentally validated a handful of targets. Aligning phenotypic characterizations with system level analysis will further provide deep mechanistic insights in order to understand complex miRNA regulatory pathways. Overall, although technological advances over the coming years will facilitate new discoveries of how non-coding RNAs impact on cellular functions, studying miRNA functions remains challenging, especially in the context of retinal degenerative diseases. Still, non-coding RNAs are key to understand comprehensively retinal functions in health and disease.

## Author Contributions

JP: writing and conceptualizing of the original draft. JP, MZ, GP, AS, and VB: writing, review, and editing. VB: funding. All authors contributed to the article and approved the submitted version.

## Conflict of Interest

The authors declare that the research was conducted in the absence of any commercial or financial relationships that could be construed as a potential conflict of interest.
